# Supplementary material for: Exploring NCATS in-house biomedical data for evidence-based drug repurposing
Source: PLoS One. 2024 Jan 25;19(1):e0289518. doi: 10.1371/journal.pone.0289518 (PMC10810548; doi:10.1371/journal.pone.0289518)
Supplement: Attachment — Submitted filename: Response to Reviewers.docx [file pone.0289518.s003.docx]

Qian Zhu, PhD

National Center for Advancing Translational Sciences (NCATS),

National Institutes of Health

9800 Medical Center Drive

Building B

Rockville, MD  20850

Work: (301) 480-7841

Dear Editors,

Thanks so much for giving us an opportunity of revising our manuscript entitled “**Exploring NCATS In-House Biomedical Data for Evidence-based Drug Repurposing**” for consideration of publication in *PLOS One*.

We would like to thank all the reviewers and the editors for their insightful comments and careful reviewing of our paper. We have revised the paper according to the reviewer comments. We clarified our responses to the reviewer comments and highlighted our changes below. Paper changes are shown in track change in the revised manuscript.

Thank you for your consideration!

Sincerely,

Qian Zhu, PhD

NCATS/NIH

Reviewer #1: The authors provide methods and supporting data illustrating how large and diverse data sets can be connected and queried with deep learning and related computational tools to generate hypotheses about compound bioactivities that may be useful in novel drug discovery. The manuscript is organized and concise and the use of three examples provides good support for the utility of the approach. The work should be of interest to those considering how to make use of large volumes of publicly available data to understand bioactivity of small molecules.

**Response**: Thanks so much for the positive comments about the importance and contribution of this work!

**Q1**: Some editing of grammar and increased clarity of statements would be beneficial.

**Response**: We thoroughly edited the whole manuscript.

**Q2**: It may also be useful for a moderate amount of additional explanations for much of the jargon used in the paper be provided as this may be unfamiliar to many readers.

**Response**: Thanks so much for the comment! We added additional explanations on those jargons.

**Q3**: Finally, it would be useful to include a brief discussion of whether the assays used in the analysis were designed to focus on a single molecular mechanism (and, hence, expect higher chemical similarity among active chemicals) or more phenotypic assays containing multiple potential molecular targets (with more chemical diversity expected).

**Response**: Thanks so much for the comment! We added additional statements to descript the assays applied in this study. “All data and detailed assay descriptions with target annotations are publicly available (<https://tripod.nih.gov/tox/pubdata/>) and PubChem database[1, 2]. Most of these assays cover targets/pathways related to nuclear receptor signaling (NR, 55.90%), stress response (SR, 11.80%), cytotoxicity (8.80%), and other toxicity-related targets/pathways (23.50%).

**Q4**: “pathophysiology-related”

**Response**: we added a statement to explain pathophysiology, “the study of abnormal changes in body functions that are the causes, consequences, or concomitants of disease processes”.

**Q5**: “Data from Tox21 for toxicology applications has been systematically preprocessed and quality control conducted (QC) [8-10],”

**Response**: we added a brief explanation about QC, verifying the data quality.

**Q6**: IDG manages two resources including the Target Central Resource Database (TCRD)
collating manage heterogeneous gene/protein datasets and Pharos providing interfaces to access data from TCRD.[14]

**Response**: we added a citation for Pharos to give more information about it, <https://academic.oup.com/nar/article/51/D1/D1405/6851109>

**Q7**: Meanwhile, the advanced computational techniques, like machine learning and
deep learning has been actively applied to learn patterns in biomedical data related to drugs and then link them up to the potential of treating alternative diseases.[24-26]”
**Response**: We rewrote this statement, “Meanwhile, the advanced computational techniques, like machine learning, deep learning has been actively applied to learn patterns in biomedical data related to drugs and then link them to support the discovery of alternative uses of drugs.”

**Q8**: “In this study, we demonstrated the use of NCATS in-house biomedical data for supporting drug repurposing.” This may be an overly strong statement. I don’t think drug repurposing was actually shown; but, rather that the approach generates relevant hypotheses.

**Response**: Thanks so much for the comment! I edited the statement accordingly, “In this study, we demonstrated the use of NCATS in-house biomedical data for generating relevant hypotheses towards drug repurposing.”
